# How does the interaction radius affect the performance of intervention on collective behavior?

**DOI:** 10.1371/journal.pone.0192738

**Published:** 2018-02-15

**Authors:** Caiyun Wang, Jing Han

**Affiliations:** 1 LSC, Academy of Mathematics and Systems Science, Chinese Academy of Sciences, Beijing 100190, China; 2 School of Mathematical Sciences, University of Chinese Academy of Sciences, Beijing 100049, China; Newcastle University, UNITED KINGDOM

## Abstract

The interaction radius *r* plays an important role in the collective behavior of many multi-agent systems because it defines the interaction network among agents. For the topic of intervention on collective behavior of multi-agent systems, does *r* also affect the intervention performance? In this paper we study whether it is easier to change the convergent heading of the group by adding some special agents (called shills) into the Vicsek model when *r* is larger (or smaller). Two kinds of shills are considered: fixed-heading shills (like leaders that never change their headings) and evolvable-heading shills (like normal agents but with carefully designed initial headings). We know that with the increase of *r*, two contradictory effects exist simultaneously: the influential area of a single shill is enlarged, but its influence strength is weakened. Which factor dominates? Through simulations and theoretical analysis we surprisingly find that *r* affects the intervention performance differently in different cases: when fixed-heading shills are placed together at the center of the group, larger *r* gives a better intervention performance; when evolvable-heading shills are placed together at the center, smaller *r* is better; when shills (either fixed-heading or evolvable-heading) are distributed evenly inside the group, the effect of *r* on the intervention performance is not significant. We believe these results will inspire the design of intervention strategies for many other multi-agent systems.

## Introduction

In recent years, collective behavior has drawn a lot of attention from scientists in many areas. It is a significant feature of self-organized multi-agent systems (MASs) where agents usually interact with each other based on local rules, i.e., each agent interacts with its neighbors. At the macroscopic level, new phenomenon will emerge in MASs which can not be found in a single agent, such as flocking of birds [1, 2], schooling of fishes [1], crowd panic [3], swarm intelligence [4], pattern formation [5, 6], synchronization [7–9], etc.

A classic example is the Vicsek model [10], a self-driven multi-agent model proposed by Vicsek et al. This model consists of autonomous agents with discrete time update rule: at each time step, velocity of all agents are updated as the vector average velocity of its neighbors with some random perturbation added and at the same time. At the same time, each agent updates its position following its velocity. The neighborhood is defined based on the interaction radius *r*, which means the distance between any two neighboring agents should be less or equal to *r*. In other words, agents only interact with their neighbors within a distance of *r*. They found an interesting phenomenon: if the density of the group is high and the noise is small, headings of all agents will converge to a same value and reach consensus. That means, with a larger interaction radius *r*, i.e., the interaction network gets more links, the group will be more likely to reach consensus.

In fact, the interaction radius has a big impact on the collective behavior of many other self-organized MASs. For example, Couzin et al. [11] found that as the alignment radius increases, agents will present different collective behavior such as swarm, torus, and consensus in a self-organizing animal group. Zúñiga and Krishnamachari [12] studied the optimal transmission radius for flooding in large scale wireless sensor networks. They found that there exists an intermediate transmission radius which minimizes the settling time for large scale wireless networks. Bagchi et al. [13] investigated the transmission radius condition that can achieve connectivity in duty-cycled wireless sensor networks. Shen et al. [14] calculated the optimal radius for caching scheme which achieves significant performance improvement in power saving, network throughput and load balance.

Another important issue is the intervention of collective behavior of MASs: if the self-organized collective behavior is not what we expect, how can we intervene the system and change the collective behavior? One way is to redesign the MAS [15–17] or put some local control in the system (pinning control) [18, 19]. The other way is nondestructive, which is called ‘soft control’ [20] proposed by Han et al. It has been successfully applied in some MASs: lead the group to converge to an expected heading of the Vicsek’s model [20, 21]; promote cooperation of multi-person prisoner’s dilemma game models [22, 23]; change the convergent opinion value in the weighted Degroot model [24]. The soft control method does not change local rules of the already-existing agents in the system, but adds one or several special agents, called shills, into the group. Shills can be redesigned and controlled, but they are treated as normal agents by normal ones. Therefore normal agents do not need to pay special attention to shills, they are not aware of the intervention. Shills can only affect their neighboring agents with normal influential power. For those MASs whose neighborhood is defined based on the interaction radius *r*, *r* not only has significant effect on the collective behavior of self-organized system, but it might also affect the soft control performance.

In this paper, we will study the problem of “**whether and how the interaction radius *r* affects the performance of soft control based on the Vicsek model**”. As we know the interaction radius *r* is an important factor for consensus of the Vicsek model. Tang et al. [25] proposed that as long as the interaction radius satisfies some conditions, the linearized Vicsek model will reach consensus with large probability. Later, Liu et al. [26] gave the similar condition for the Vicsek model. Furthermore, Chen et al. [27] studied the smallest interaction radius for the synchronization behavior of the Vicsek model.

As a starting point, this paper is based on specific cases of the Vicsek model: *n* normal agents with initial heading *θ*_0_ are randomly placed inside an *M* × *M* periodic boundary square area. It means the group has reached consensus (heading *θ*_0_). Now the intervention purpose is to change the consensus heading. Using the soft control idea, one or several shills with initial heading *θ*_*s*_ are added into the group. Two scenarios are considered: (i) ***evolvable-heading-shill***, which means the shill will be affected by its neighbors and its heading is updated following the same update rule as normal agents, so the group will converge to *θ*′ which will be on average inside the interval between *θ*_0_ and *θ*_*s*_ (0 = *θ*_0_ < avg. *θ*′ *θ* < *θ*_*s*_) if the population is suitably large [26]. In this scenario, changing the interaction radius *r* will change the convergent heading value of the system. Therefore, we use the change of convergent heading of normal agents Δ*θ* (Δ*θ* = *θ*′ − *θ*_0_) to measure the soft control performance. Larger Δ*θ* means better soft control performance; (ii) ***fixed-heading-shill***, which means the shill’s heading is fixed during evolution. They are persistent, like the leader in [28]. Therefore, the group will eventually converge to *θ*_*s*_. In this scenario, changing the interaction radius *r* will not change the convergent heading value but it will change the number of convergent time steps (relaxation time) *T*. So we use *T* to measure the soft control performance. Smaller *T* means better soft control performance. In each of these two scenarios, two strategies are considered: (i) ***centered***, which means shills are staying together and initially placed at the center of the square, so they are neighbors of each other; (ii) ***distributed***, which means shills are regularly distributed inside the square, so they are separated. Therefore, we have 4 combinations of different scenarios and strategies. Then our problem of “how does the interaction radius affect the soft control performance” is split into two specific sub-problems:

**(1) what is the optimal interaction radius that can maximize the change of convergent heading of normal agents** Δ*θ* = *θ*′ − *θ*_0_, **i**.**e**., $r_{optimal}=\underset{r}{arg max}\Delta \theta \left(r\right)$, **in the ‘*evolvable-heading-shill*’ scenario for both strategies**?

**(2) what is the optimal interaction radius that shills can lead normal agents to converge to *θ*_*s*_ with the smallest number of time steps**, **i**.**e**., $r_{optimal}=\underset{r}{arg min}T\left(r\right)$, **in the ‘*fixed-heading-shill*’ scenario for both strategies**?

We find answers to the above two problems through simulations and some related theoretical analysis: for the *centered fixed-heading-shill* case, larger *r* leads to better soft control performance (smaller *T*); for the *centered evolvable-heading-shill* case, smaller *r* gives better soft control performance (larger Δ*θ*); for the *distributed* strategy (both in the *fixed-heading-shill* and the *evolvable-heading-shill* scenarios), the effect of *r* on the control performance is not significant. On the other hand, we also find some interesting results by comparing the *distributed* strategy and the *centered* strategy: for the *fixed-heading-shill* scenario, the *distributed* strategy outperforms the *centered* strategy; while for the *evolvable-heading-shill* scenario, the *centered* strategy outperforms the *distributed* strategy because of the ‘reciprocal team’ effect—the neighboring evolvable-heading shills support and interact with each other.

There are some related studies about intervention on flocking models. Kyriakopoulos et al. [29] studied the response of flocks, described as hydrodynamic equation which contains the Vicsk model, to a small homogeneous external field of amplitude *h*. The function of external field is like the *fixed-heading* shill, if the external field is constructed by continuously adding the same number of *fixed-heading* shills to the neighborhood of every normal agent. On the other hand, the *fixed-heading* shill with velocity *v* > 0 in our approach can be regarded as a moving local external field. However, our *fixed-heading* shills methods is different to external field in general. Shills are more like an agent than external field because they are moving and their influential area is decided by the interaction neighborhood radius *r* as same as that of all normal agents. Even though in the *distributed* strategy, normal agents usually do not have the same number of neighboring *fixed-heading* shills, especially when *v* > 0. That makes the ‘external field’ constructed by *fixed-heading* shills very complicated. Pearce et al. [30] considered some leaders (informed individuals [31]), who update their headings by taking average of the heading of their neighbors and with a biased specific direction, based on the Vicsek model. They studied how the flock responses to the leadership. Their approach has some essential differences with our paper. First, these leaders who are biased to turn toward a specific angle are different from our shills: *fixed-heading* shills always head to a fixed desired direction; *evolvable-heading* shills follow the same updating rule as normal agents but with well-designed initial headings. Second, [30] considered the Vicsek model with a fixed interaction radius *r* = 1; while in this paper a wider range of *r* is investigated to study the impact of *r* on the control performance. Third, because the dynamic of leaders is different from the shills, the conclusions on linearity are different: [30] found that the group shows a linear response to leadership; while in this paper we will show that the response of the group to shills is not linear.

## Methods

### The basic model

Vicsek et al. proposed a self-propelled multi-agent model in 1995 [10]. Suppose there are *n* agents (***x***_***i***_(*t*), *θ*_*i*_(*t*)), labelled from 1 to *n*, are placed inside an *M* × *M* periodic square area, where ***x***_***i***_(*t*) denotes the position of agent *i* at time *t* and *θ*_*i*_(*t*) denotes the heading of agent *i* at time *t*. The velocity of agent *i* at time *t* is defined by ***v***_*i*_(*t*) = (*v* cos(*θ*_*i*_(*t*)), *v* sin(*θ*_*i*_(*t*))) is constructed to have an constant absolute value *v* and a heading given by the angle *θ*_*i*_(*t*) ∈ (−*π*, *π*]. The neighborhood of agent *i* is defined as

$$\begin{array}{c} N_{i}\left(t\right)=\{j|∥x_{j}\left(t\right)-x_{i}\left(t\right)∥\leq r,j=1,\cdots ,n\}, \end{array}$$ (1)

where *r* is a given interaction radius. This means two agents are neighbors if the distance between them is not larger than *r*.

For any agent *i*, its position and heading are updated by Eqs (2)–(3) below:

$$\begin{array}{c} x_{i}\left(t+1\right)=x_{i}\left(t\right)+v_{i}\left(t\right), \end{array}$$ (2)

$$\begin{array}{c} \theta_{i}\left(t+1\right)={\langle \theta \left(t\right)\rangle }_{r}+\delta_{i}\left(t\right), \end{array}$$ (3)

where 〈*θ*(*t*)〉_*r*_ denotes the average direction of the velocities of neighbors of agent *i*. In other words, 〈*θ*(*t*)〉_*r*_ has the following form:

$$\begin{array}{c} {\langle \theta \left(t\right)\rangle }_{r}=\text{arctan}\left(\frac{\sum_{j\in N_{i}\left(t\right)}\text{sin}\left(\theta_{j}\left(t\right)\right)}{\sum_{j\in N_{i}\left(t\right)}\text{cos}\left(\theta_{j}\left(t\right)\right)}\right) \end{array}$$ (4)

with some necessary regulations reflecting heading of (−*π*, *π*]. In Eq (3), *δ*_*i*_(*t*) represents noise and it is a random number chosen with a uniform probability from the interval [−*η*/2, *η*/2].

Here is the evolution process of the Vicsek model: after the initial heading and the position of each agent are given, all agents update their headings using Eqs (1)–(4) at every discrete time step *t* = 1, 2, ⋯. The simulation results in Vicsek’s paper [10] show that if the density is large and noise is small, the group will reach consensus. In Liu’s paper [26], they prove that without noise, the Vicsek model will almost surely reach consensus for any *r* > 0 and *v* > 0.

### Model with shills

Suppose the group has reach consensus (heading *θ*_0_). Now we want to change the consensus heading. Then we add *l* (*l* ≥ 1) shills with heading *θ*_*s*_ into the group and reset the clock as *t* = 0. Therefore we now have *θ*_*i*_(0) = *θ*_0_ for *i* = 1, 2, ⋯, *n*. Because normal agents regard shills as normal ones while updating their headings, the neighborhood of all agents should be redefined as follows:

$$\begin{array}{c} N_{i}\left(t\right)=\{j|∥x_{j}\left(t\right)-x_{i}\left(t\right)∥\leq r,j=1,\cdots ,\tilde{n}\}\;\text{for}\;i=1,\cdots ,\tilde{n}, \end{array}$$ (5)

where $\tilde{n}=n+l$ is the total number of agents, agent 1, 2, ⋯, *n* are normal agents, agent *n* + 1, *n* + 2, ⋯, *n* + *l* are shills. The absolute value of the velocities of shills is also set to be *v* here. After adding shills into the system, normal agents will evolve following Eqs (2)–(5).

### The effect of interaction radius *r*

Since the neighborhood is decided based on *r*, the neighborhood graph might be different if *r* is different. Therefore, *r* also has significant impact on the effect of soft control. For any *r*, two factors describe how much the influence of one shill bring to the system: the influential area *S* and the influence strength *P*. The influential area *S* is the number of normal agents which are affected by one shill, i.e., *S* = *πr*^2^ *ρ*_*n*_, where *ρ*_*n*_ is the density of normal agents. The influence strength *P* is the influence that one shill puts on its neighboring agents. The measurement for *P* is much more complicate than *S*, because from the second step, headings of normal agents might not be the same, and headings of *evolvable-heading* shills are no longer *θ*_*s*_. So for simplicity, we consider *P* only for the first step influence in an ideal case: suppose the initial heading *θ*_0_ of normal agents neighboring to the shill is *zero*, then we have $P=\arctan(\frac{\sin\theta_{s}}{S+\cos\theta_{s}})$. Lager *P* means more powerful influence strength that one shill puts on its neighboring normal agents. Apparently, *P* decreases with the increase of *S*. From this simplified analysis, there is a tradeoff between *P* and *S*.

Smaller (or larger) interaction radius *r* has both the negative and positive effects when we consider of how much influence of one shill brings to the group:

- The **positive effect** for smaller *r*: the influence that one shill puts on its neighboring agents *P* is larger. With the increase of *r*, *S* becomes larger which makes *P* smaller.
- The **negative effect** for smaller *r*: the influence area of one shill *S* is smaller. This means there are less normal agents directly influenced by one shill.

Therefore, if the positive effect of smaller *r* dominates, smaller *r* will give better soft control performance. Otherwise, larger *r* will give better performance. But which effect dominates? And in what circumstances?

**Fig 1:**
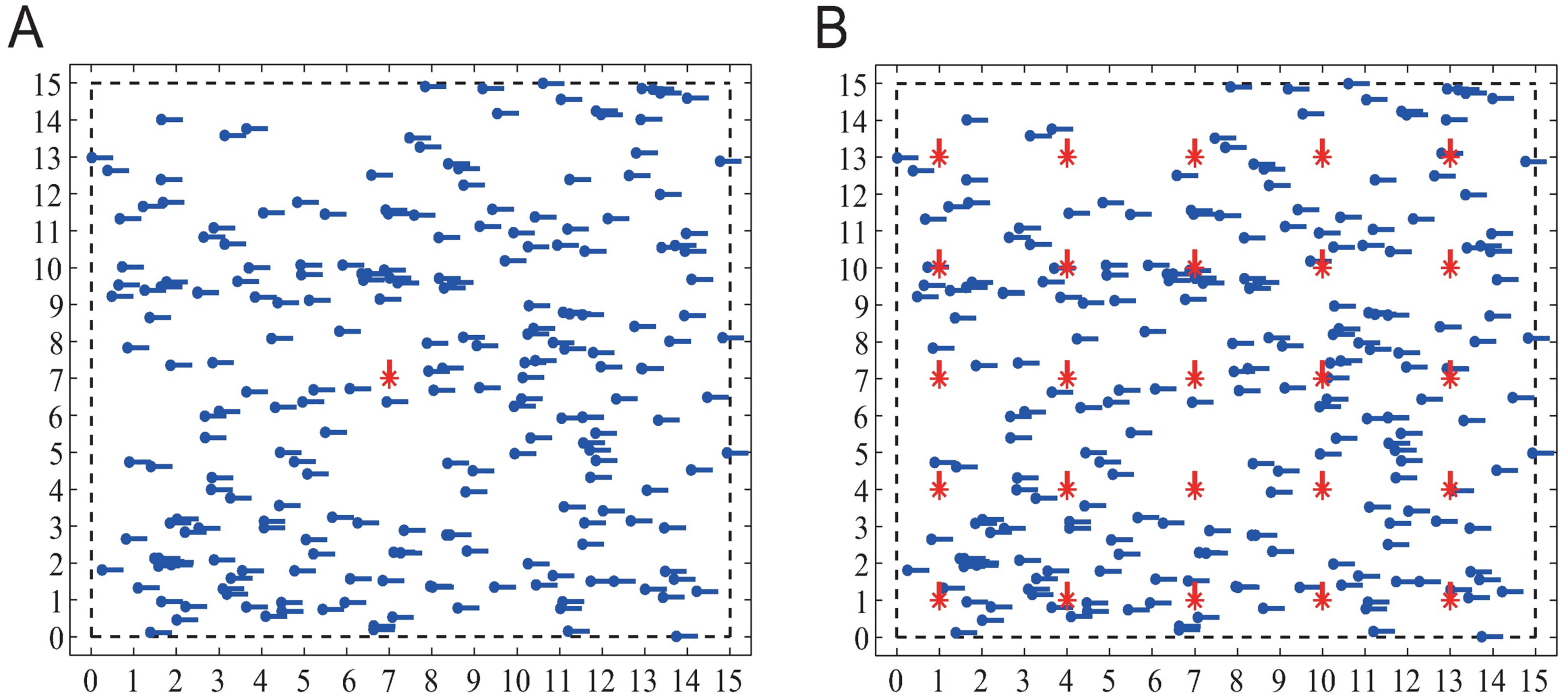
Examples of initial positions of adding 25 shills into a group of 225 normal agents inside a 15 × 15 square. Blue dots represent normal agents. Red stars represent shills. Short lines connected to the blue dots or red stars represent their headings. Here initial headings of all normal agents are set as *zero* and initial headings of shills are *π*/2. (A) *Centered* shills; (B) *Distributed* shills.

To answer above questions, we consider our study in the circumstance of two different soft control strategies (*centered* and *distributed*) in two scenarios (*fixed-heading-shill* and *evolvable-heading-shill*):

- ***Centered***: *l* shills are initially centered inside an *M* × *M* periodic boundary square. This means all shills are initially located in the same position, the center point of the square (example is Fig 1a).
- ***Distributed***: *l* shills are initially regularly distributed inside an *M* × *M* periodic boundary square (example is Fig 1b). In this way, the *M* × *M* square is evenly divided into *l* small squares, where each of them initially locates one shill. These shills will not be neighbors of each other if *r* is not large enough.

When *l* shills are initially placed at the center, they will have strong influence on their neighboring normal agents. These shills act like a ‘core’, the neighboring normal agents will align with the ‘core’ quickly and become ‘virtual shills’ in some sense. The ‘core’ and the ‘virtual shills’ form an ‘alliance’ in the center. When *l* shills are distributed in the area, their influence is evenly distributed among the group, the ‘core’ effect of each shill is much weaker.

Two scenarios are:

- ***Fixed-heading-shill***: *l* *fixed-heading* shills with heading *θ*_*s*_ are added into an *M* × *M* periodic boundary square. It means headings of shills are not affected by their neighbors. They are always equal to *θ*_*s*_.
- ***Evolvable-heading-shill***: *l* *evolvable-heading* shills with initial heading *θ*_*s*_ are added into an *M* × *M* periodic boundary square. It means headings of shills are initialized as *θ*_*s*_, and then they will be updated following Eqs (2)–(5) without noise, just like normal agents.

If *l* *evolvable-heading* shills are initially distributed inside the group, each of them will be easily affected by its neighboring normal agents and lose the shill effect within the first few steps. But if they are initially placed together at the center, they will interact with each other and become a reciprocal team, which forms a strong ‘alliance’ around them. We call this the ‘**reciprocal team**’ effect, which becomes significant with the increase of the number of neighboring *evolvable-heading* shills in the team. It cannot be found in the *fixed-heading-shill* scenario, because these shills do not interact with each other.

## Results and discussions

To study effects of interaction radius on the soft control performance based on the Vicsek model, a number of simulations are performed for four combinations of two strategies (*centered* and *distributed*) in two scenarios (*fixed-heading-shill* and *evolvable-heading-shill*). And then we extend this study to the linearized Vicsek model and the non-periodic boundary model cases. Some related discussions and theoretical analysis are given.

Here are settings of simulations in this section: *v* = 0.03 which is the same as the setting of [10]; *η* = 0.02; *θ*_0_ = 0. *n* normal agents with initial heading *θ*_0_ are randomly placed inside the *M* × *M* square. Soft control performance is measured as the average of 100 runs on random position distributions of normal agents. For each simulation, we add *l* shills into the system. For the *centered* strategy, all *l* shills are initially placed at the same position (example is Fig 1a with *M* = 15 and *l* = 25); for the *distributed* strategy, each shill is initially placed at the center of every $\frac{M}{\sqrt{l}}\times \frac{M}{\sqrt{l}}$ square (example is Fig 1b with *M* = 15 and *l* = 25). In [10], Vicsek et al. proposed an order parameter $\phi \left(t\right)=\frac{1}{\tilde{n}v}|\sum_{i=1}^{\tilde{n}}v_{i}\left(t\right)|$, where ***v***_*i*_(*t*) = (*v* cos *θ*_*i*_(*t*), *v* sin *θ*_*i*_(*t*)), to measure the consensus level. In our simulations, the system is regarded as consensus when 1 − *ϕ*(*t*) ≤ 10^−4^, then the simulation is terminated.

### *Fixed-heading-shill* scenario

In this subsection, we add *l* *fixed-heading* shills with heading *θ*_*s*_ into an *M* × *M* square with periodic boundary. The number of convergent time steps *T* is used to measure the soft control performance. We find that *r* has big impact on the value of *T* in the following simulations.

**Fig 2:**
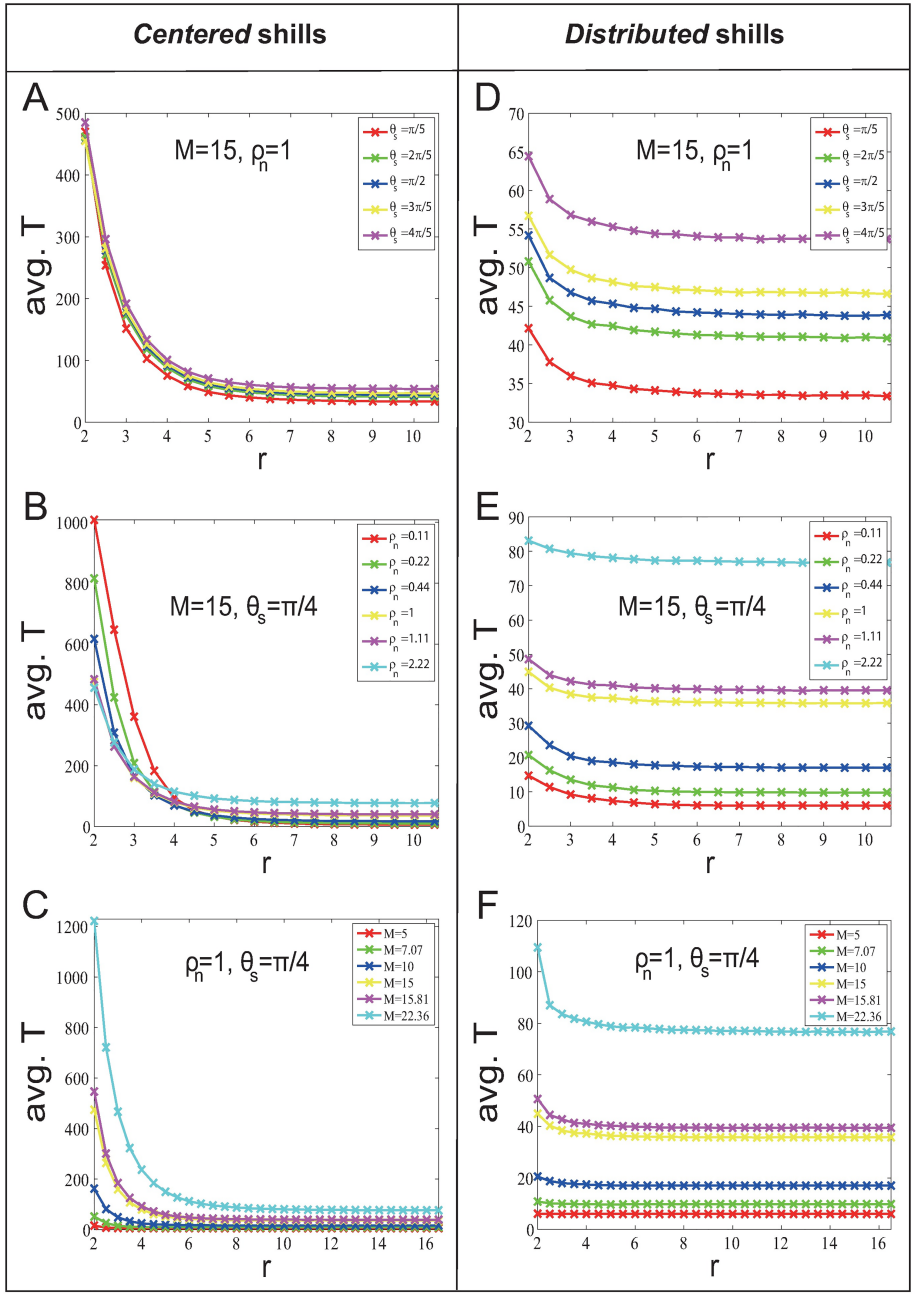
Soft control performance (avg. *T*) for different *θ*_*s*_, *ρ*_*n*_ and *M* in the *fixed-heading-shill* scenario with *l* = 25.

Fig 2 shows avg. *T* for different *θ*_*s*_, *M* and *ρ*_*n*_ with *η* = 0.02, and has the following results (results are also hold for *η* = 0.1 (see S1 Fig for details)):

- For the *centered* strategy, larger *r* gives better soft control performance (smaller avg. *T*).
  Although smaller *r* has positive effect, there is a big delay of shill influence for normal agents located far away from shills. Therefore it needs more time steps to spread the shill effect to all normal agents. In this case, the negative effect overcomes the positive effect of smaller *r*, so larger *r* gives better soft control performance.
- For the *distributed* strategy, the effect of *r* on the soft control performance is not significant.
  In this case, the positive effect and the negative effect of smaller *r* are balanced in some way, so *T* varies very little while *r* changes. The mean-field analysis is given in S1 Appendix.

**Fig 3:**
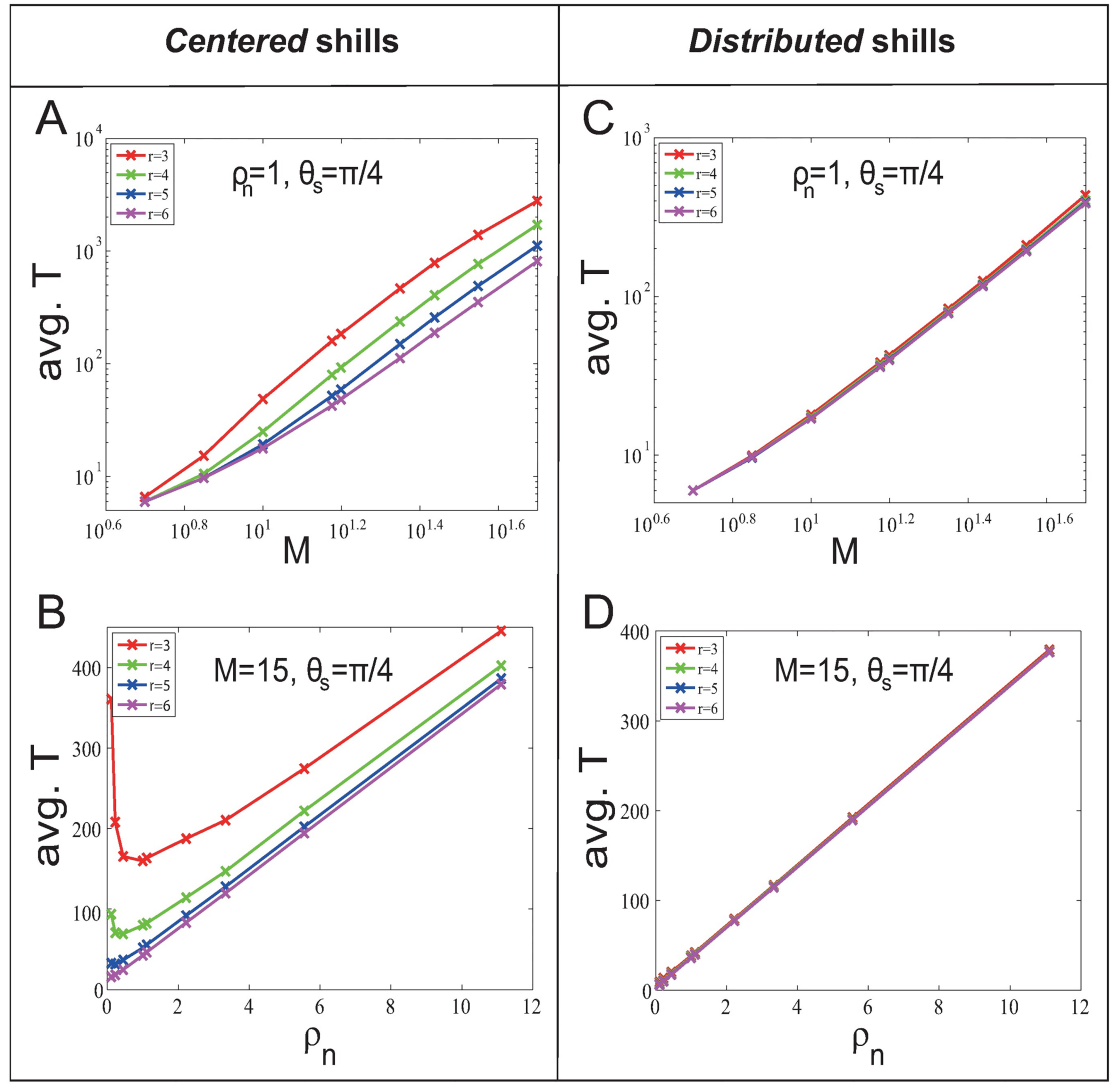
System size effect on soft control performance (avg. *T*) in the *fixed-heading-shill* scenario with *l* = 25. The system size is *n* = *ρ*_*n*_ *M*^2^. (A) and (C) show how soft control performance (avg. *T*) change when *n* increases (by increasing *M* with fixed *ρ*_*n*_); (B) and (D) show how soft control performance (avg. *T*) change when *n* increases (by increasing *ρ*_*n*_ with fixed *M*).

Then we investigate the finite system size effect. The system size is the number of normal agents *n* (*n* = *ρ*_*n*_ *M*^2^). Fig 3a and 3c show that avg. *T* and *M* approximately obey positive power function regardless of *r*. Fig 3b and 3d show that in general the avg. *T* increases with the increase of *ρ*_*n*_. This is because more normal agents are needed to be influenced with the increase of *ρ*_*n*_. But in Fig 3b, there is a special phenomenon when *r* and *ρ*_*n*_ are small: avg. *T* decreases with the increase of *ρ*_*n*_. This is because the neighborhood graph is too sparse that it takes more time steps for the shill influence to spread to all normal agents.

**Fig 4:**
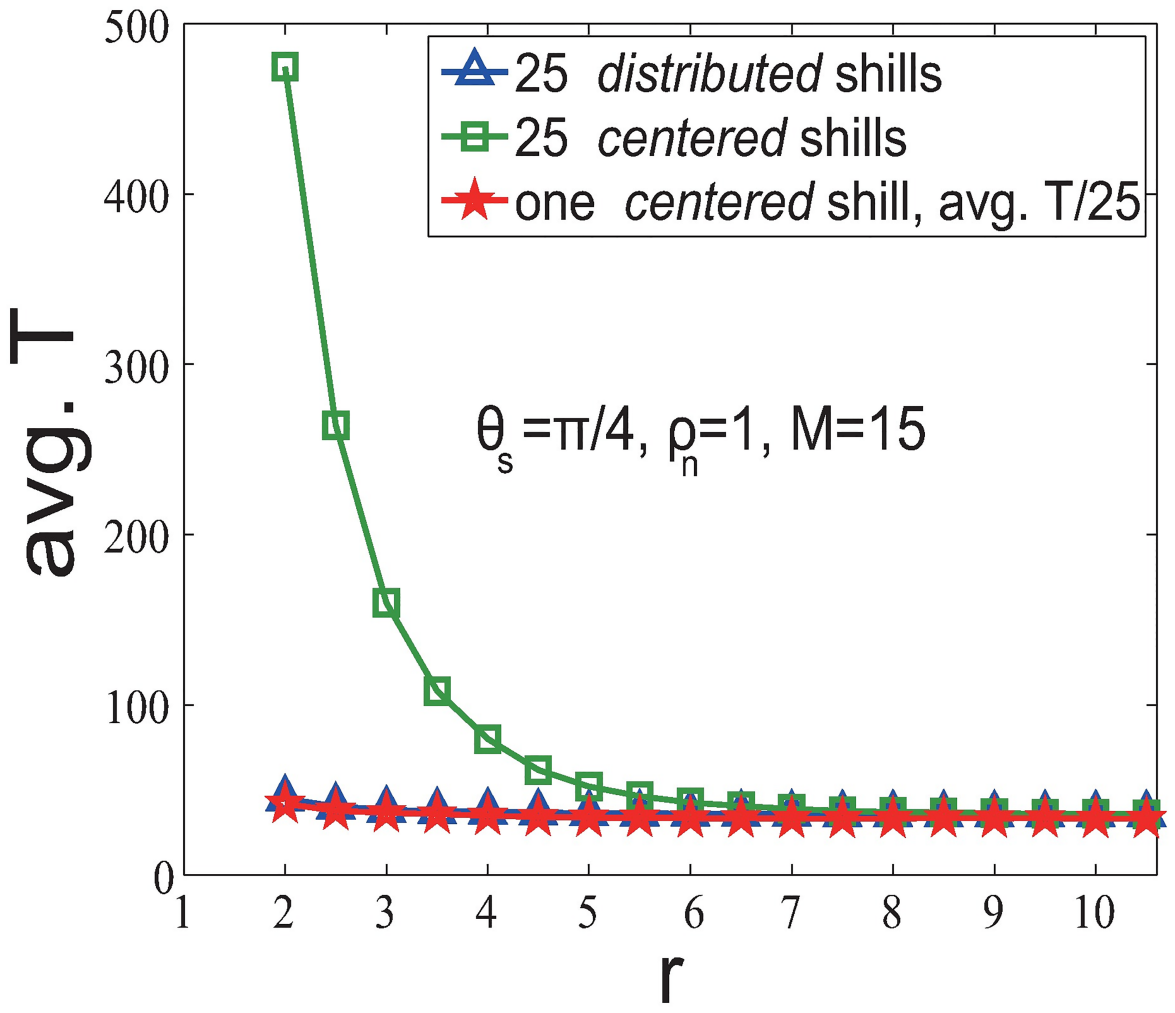
Comparisons of different soft control strategies in the *fixed-heading-shill* scenario.

Furthermore, in Fig 4 we compare the performance between *centered* and *distributed* strategies, and investigate whether adding *l* shills can improve the soft control performance by the factor of *l* comparing to the case of adding one shill. We find the following results with *θ*_*s*_ = *π*/4 (results are also hold for other *θ*_*s*_ (see S2 Fig for details)):

- *Distributed* strategy (blue line) outperforms *centered* strategy (green line).
- 25 *centered* shills (green line) do not enhance 25 times of the soft control performance of one single shill (red line).
  We compare the number of convergent time steps of adding 25 *centered* shills with 1/25 of the number of convergent time steps of adding one shill. We find that the number of convergent time steps of adding one shill is smaller than 25 times of the number of convergent time steps of adding 25 *centered* shills. Therefore, the performance of *l* *fixed-heading* shills is less than the linear superposition.

### *Evolvable-heading-shill* scenario

In this subsection, we add *l* *evolvable-heading* shills with initial heading *θ*_*s*_ into an *M* × *M* square with periodic boundary. These shills will be affected by neighboring normal agents and update their headings during evolution like normal ones but without noise. Given *θ*_0_ = 0 and *θ*_0_ < *θ*_*s*_ in our simulations, heading of agents in the group will converge to value *θ*′ (0 < avg. *θ*′ < *θ*_*s*_) if the population is suitably large [26]. Therefore, the change of convergent heading of normal agents Δ*θ* (Δ*θ* = *θ*′ − *θ*_0_ = *θ*′) is used to measure the soft control performance. Larger Δ*θ* means better performance of soft control. We find that *r* has big impact on the value of Δ*θ* in the following simulations.

**Fig 5:**
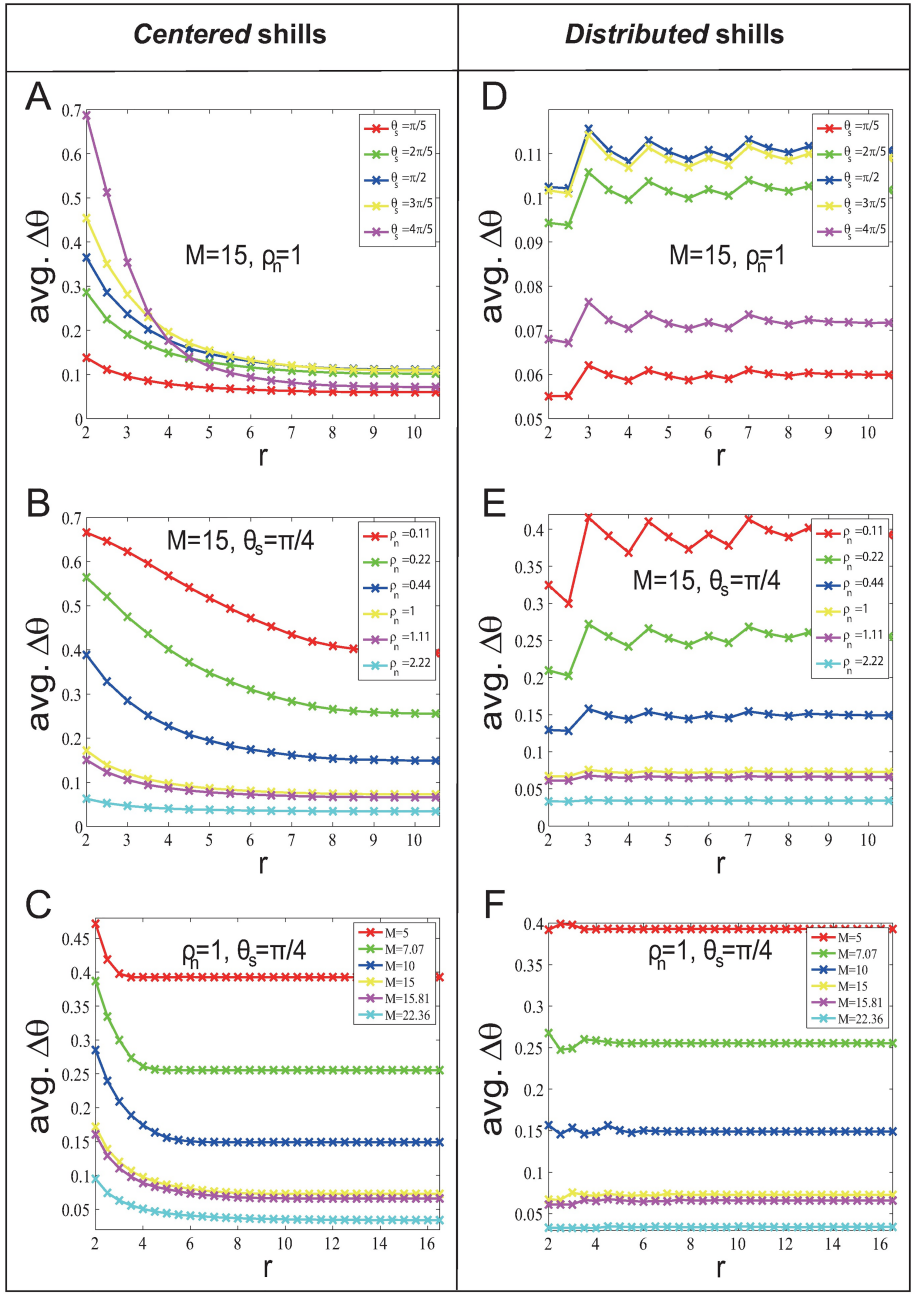
Soft control performance (avg. Δ*θ*) for different *θ*_*s*_, *M* and *ρ*_*n*_ in the *evolvable-heading-shill* scenario with *l* = 25.

Fig 5 shows avg. Δ*θ* for different *θ*_*s*_, *M* and *ρ*_*n*_ with *η* = 0.02, and has the following results (results are also hold for *η* = 0.1 (see S1 Fig for details)):

- For the *centered* strategy, smaller *r* gives better soft control performance (larger Δ*θ*).
  This is unlike the result in the *fixed-heading-shill* scenario. It is because the smaller *r* gives stronger ‘reciprocal team’ effect. While in the *fixed-heading-shill* scenario, there is no ‘reciprocal team’ effect.
- For the *distributed* strategy, the effect of *r* on the soft control performance is not significant.
  This is similar to what we have found in the *fixed-heading-shill* scenario. It is worth mentioning that for *θ*_*s*_ > *π*/2, increasing *θ*_*s*_ decreases Δ*θ*. This is caused by the nature of the updating rule of agents—each agent updates its heading by taking average direction of the velocities of its neighbors. The mean-field analysis is given in S1 Appendix.

**Fig 6:**
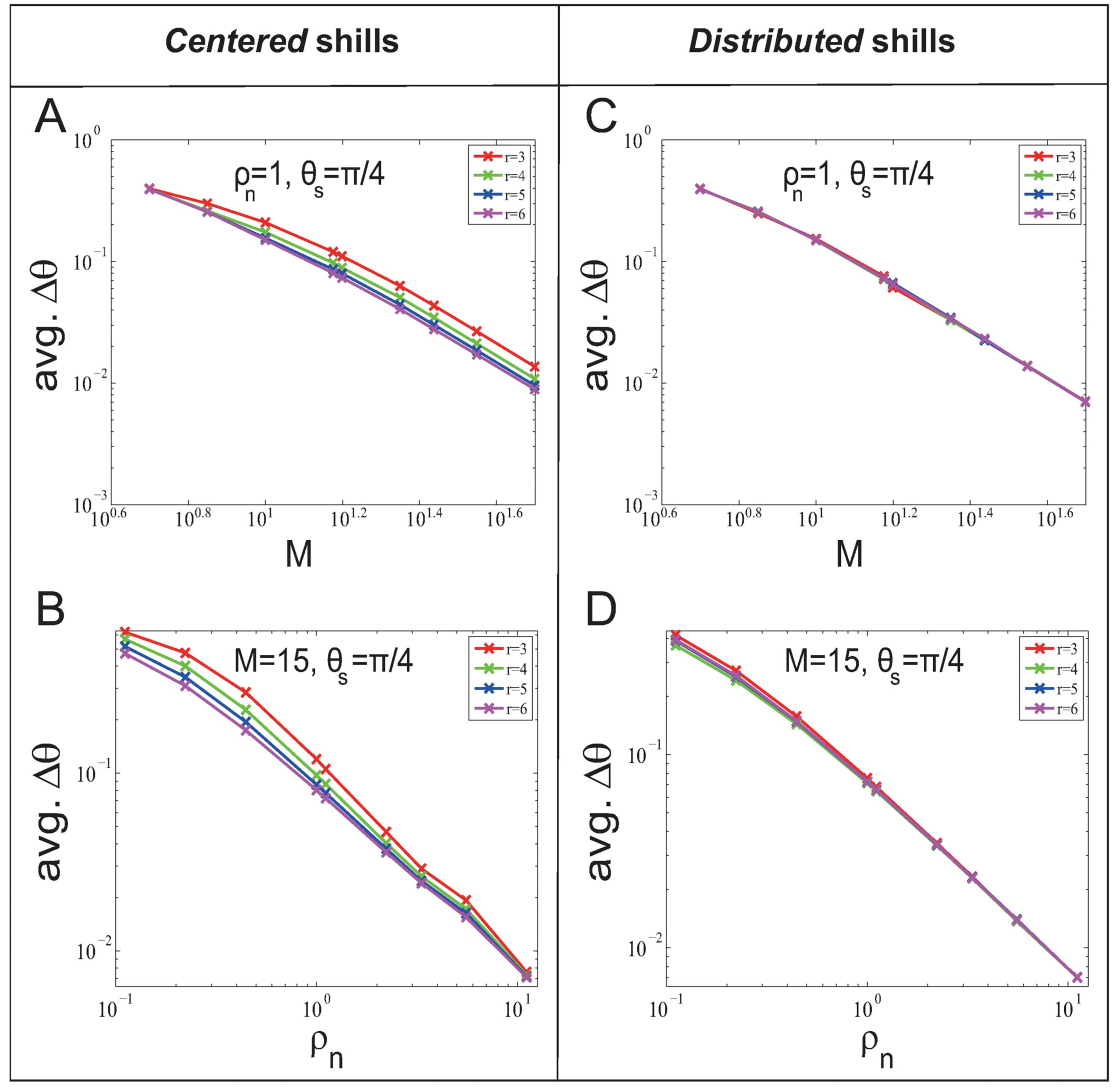
System size effect on soft control performance (avg. *T*) in the *evolvable-heading-shill* scenario with *l* = 25. (A) and (C) show how soft control performance (avg. Δ*θ*) change when *n* increases (by increasing *M* with fixed *ρ*_*n*_); (B) and (D) show how soft control performance (avg. Δ*θ*) change when *n* increases (by increasing *ρ*_*n*_ with fixed *M*).

Then we investigate the finite system size effect. The system size is *n* = *ρ*_*n*_ *M*^2^. Fig 6a and 6c show that avg. Δ*θ* and *M* approximately obey negative power function regardless of *r*. Fig 6b and 6d show that avg. *T* and *ρ*_*n*_ approximately obey negative power function regardless of *r*.

**Fig 7:**
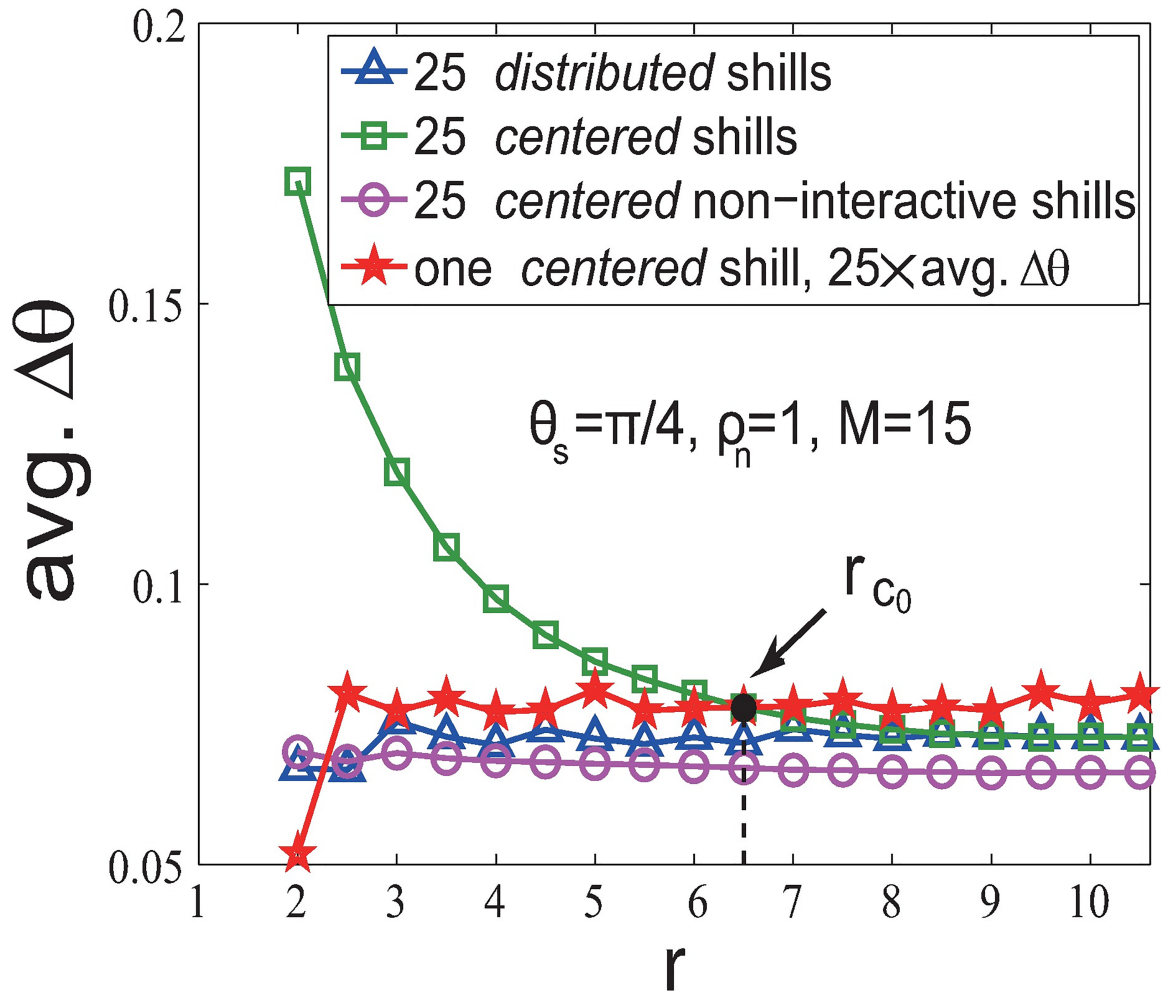
Comparisons of different soft control strategies in the *evolvable-heading-shill* scenario with *ρ*_*n*_ = 1 and *θ*_*s*_ = *π*/4.

Again, to compare the soft control performance between *centered* and *distributed* strategies, study the linear superposition property, and demonstrate the importance of ‘reciprocal team’ effect, we compare the performance (Δ*θ*) for four situations in Fig 7 with *θ*_*s*_ = *π*/4 (results are also hold for other *θ*_*s*_ (see S2 Fig for details)):

- *Centered* strategy (green line) outperforms *distributed* strategy (blue line).
  It is because the significant ‘reciprocal team’ effect strongly strengthen the shill effect for the *centered* strategy.
- *Centered* (interactive) shills (green line) outperform *centered* non-interactive shills (purple line).
  This pattern shows the importance of the ‘reciprocal team’ effect. Interactive shills have the ‘reciprocal team’ effect, while non-interactive shills do not. Without the ‘reciprocal team’ effect, the performance (purple line) is much worse (than the green line) and the pattern is more like the single shill case (red line). Therefore, the interactions among *evolvable-heading* shills significantly enhance the soft control power.
- 25 *centered* shills (green line) v.s. one single shill (red line) for small *r*.
  Unlike the result in the *fixed-heading-shill* scenario, we find that the performance of adding 25 *centered* shills is much better than 25 times of adding one *centered* shill when *r* is smaller than a critical point *r*_*c*_0__. This can be regarded as a ‘1 + 1 > 2’ phenomenon, and shows how important it is for shills to stay together and support each other when their headings are evolvable (not persistent), especially when *r* is small. This means that the significant ‘reciprocal team’ effect brings in a nonlinear enhancement for the soft control power especially when *r* < *r*_*c*_0__. When *r* > *r*_*c*_0__, increasing numbers of neighboring normal agents of shills will strongly dilute the shill influence in first few steps. Notice that the critical point *r*_*c*_0__ varies with different initial heading of shills (see S3 Fig for details).

### Extension to the linearized Vicsek model

Jadbabaie et al. [28] proposed the linearized Vicsek model by changing Eq (4). For any agent *i*, its heading is updated as the average heading of its neighboring agents:

$${\langle \theta \left(t\right)\rangle }_{r}=\frac{1}{\left|N_{i}\left(t\right)\right|}\sum_{j\in N_{i}\left(t\right)}\theta_{j}\left(t\right),$$ (6)

where |*N*_*i*_(*t*)| is the number of agent *i*’s neighbors.

The simulation results for the linearized Vicsek model is similar for these of the Vicsek model and we give theoretical analysis for these patterns (see S2 Appendix for details).

### Extension to non-periodic boundary model

**Fig 8:**
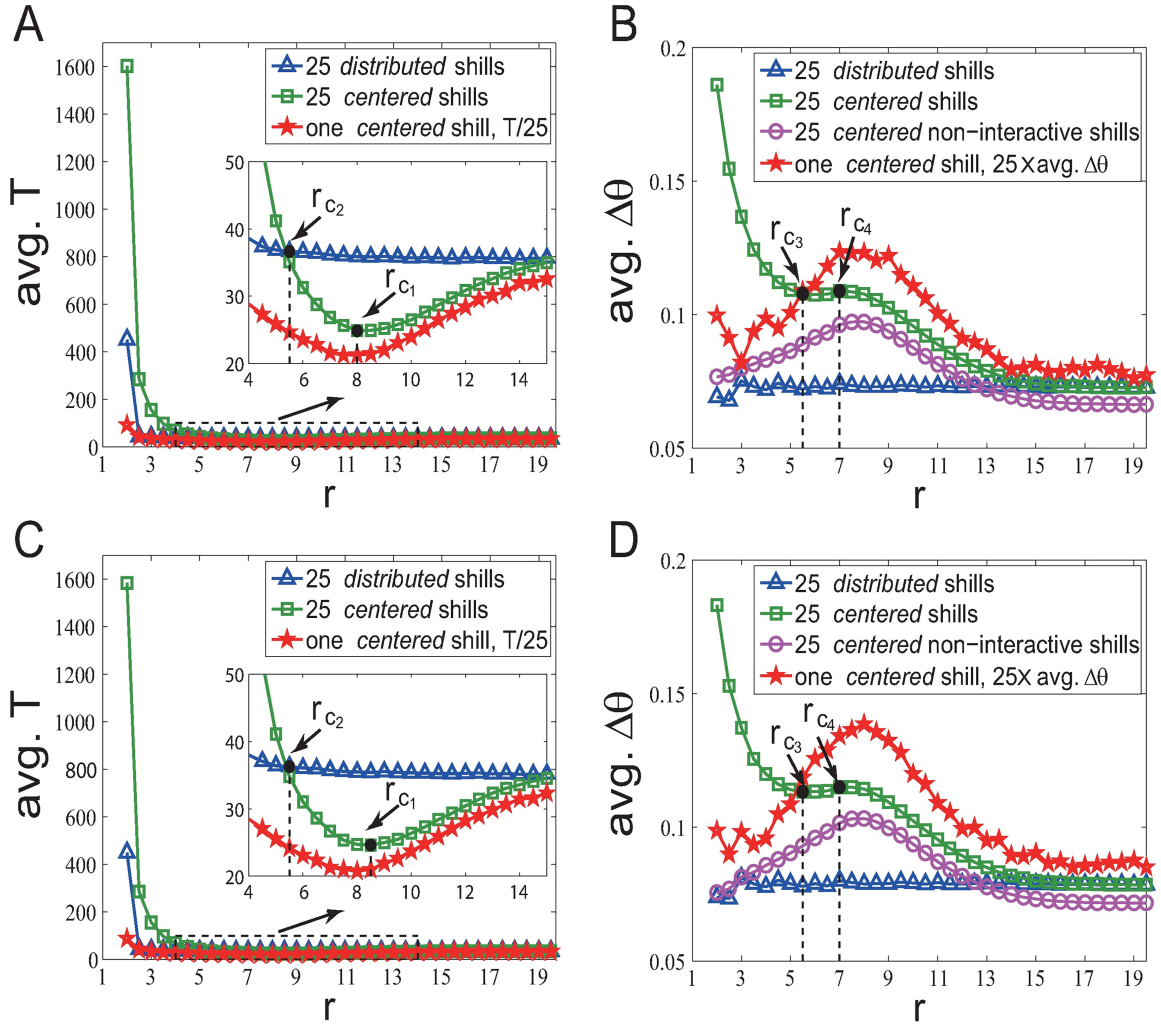
Soft control performance of different strategies in different scenarios based on two different models (the Vicsek model and the linearized Vicsek model) with non-periodic boundary. *ρ*_*n*_ = 1, *M* = 15, *l* = 25, *v* = 0 and *η* = 0.02. (A) *Fixed-heading-shill* scenario for the Vicsek model. (B) *Evolvable-heading-shill* scenario for the Vicsek model. (C) *Fixed-heading-shill* scenario for the linearized Vicsek model. (D) *Evolvable-heading-shill* scenario for the linearized Vicsek model.

What we have studied above are for periodic boundary cases (based on both the Vicsek model and the linearized Vicsek model). For non-periodic boundary cases, agents located near the boundary have less neighboring agents. Our earlier conference paper [32] has shown some simulation results for the non-periodic boundary Vicsek model with *zero* absolute velocity for *n* = 200 and *l* = 25. To be consistent with the parameter settings of this paper, we re-do simulations for the non-periodic boundary Vicsek model with *zero* velocities and noise (Fig 8a and 8b) and the non-periodic boundary linearized Vicsek model with *v* = 0 (Fig 8c and 8d).

Patterns for non-periodic boundary cases are similar but not exactly the same as patterns that we have found in the periodic boundary cases because of the boundary effect. But they share similar patterns in many ways: (i) for the *centered fixed-heading-shill* case, larger *r* gives better soft control performance when *r* is smaller than a relatively large value (*r* < *r*_*c*_1__); (ii) for the *centered evolvable-heading-shill* case, smaller *r* gives better soft control performance except a short interval of *r* in the middle (*r*_*c*_3__ < *r* < *r*_*c*_4__); (iii) for the *distributed* strategy (in both the *fixed-heading-shill* and the *evolvable-heading-shill* scenarios), the effect of *r* on the soft control performance is not significant; (iv) the *centered* strategy outperforms the *distributed* strategy in the *evolvable-heading-shill* scenario; the *distributed* strategy outperforms the *centered* strategy for small *r* (*r* < *r*_*c*_2__) in the *fixed-heading-shill* scenario. The ‘reciprocal team’ effect are also found.

## Conclusions

**Fig 9:**
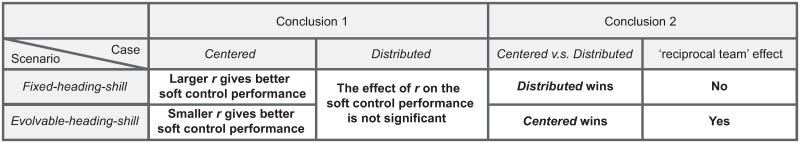
Main conclusions.

Based on a classic multi-agent system model, the Vicsek model, we study the effect of the interaction radius on the soft control performance. Then we extend the approach to the linearized Vicsek model and the non-periodic boundary model cases. We consider two different soft control strategies (*centered* and *distributed*) in two scenarios (*fixed-heading-shill* or *evolvable-heading-shill*). We obtain the following results (see Fig 9 for details) through simulations and analysis:

1. Answer to the question of “how does the interaction radius affect the performance of soft control based on the Vicsek model” (with periodic boundary) is shown as conclusion 1 in Fig 9.
  To explain these results, we analyze and discuss the positive and negative effects of the smaller/larger interaction radius *r* on the soft control performance. These two effects are competitive: the positive effect of smaller *r*—there will be fewer connections among normal agents, so the strength of influence that a shill puts on a neighboring normal agent will be stronger since the normal agent has fewer neighbors; the negative effect of smaller *r*—the influential circle of a shill is smaller, so fewer normal agents are directly influenced by a single shill. These two factors (the shill’s influential area and the shill’s influence strength) are contradictory: as one falls, another rises. Therefore, there is a tradeoff between the negative and positive effects of smaller/larger *r* on the soft control performance. The optimal value of *r* is different for different strategies and scenarios. And we give mean-field analysis for the *distributed* strategy.
2. Another interesting question is: should we place shills together at the center or distribute them evenly among the group while considering different *r*? We find the following answers regardless of *r* (shown as conclusion 2 in Fig 9): **(i) for the *fixed-heading-shill* scenario, the *distributed* strategy outperforms the *centered* strategy; (ii) for the *evolvable-heading-shill* scenario, the *centered* strategy outperforms the *distributed* strategy**. *Fixed-heading* shills are persistent individuals. They affect their neighbors but never be affected. While *evolvable-heading* shills are not that ‘strong-minded’. They interact with their neighbors and will be affected by them. Therefore, it will make a big difference if *evolvable-heading* shills are staying together to support each other—this is called the ‘reciprocal team’ effect, which brings in a nonlinear enhancement for the soft control power. We can see that the soft control performance is much worse in the *centered* non-interactive *evolvable-heading-shill* case where interactions among *evolvable-heading* shills are removed. This interesting phenomenon can be concluded from anther aspect: the performance of *l* *fixed-heading* shills is worse than linear superposition; the performance of *l* *evolvable-heading* shills is better than linear superposition when *r* is small.
3. We extend our study to the linearized Vicsek model, and we find similar results. Besides simulations, we also give mathematical analysis to these patterns in the *evolvable-heading-shill* scenario with *v* = 0 and prove that these results are true for *v* > 0 under some conditions. We also extend our study to the non-periodic boundary model and find its patterns are similar but not exactly the same as patterns that we have found in the periodic boundary cases because of the boundary effect. The ‘reciprocal team’ effect are also found in non-periodic boundary cases.

This paper explores a new question which is about the relationship between the interaction radius and the intervention performance in different cases. It will help intervention on collective behavior of MASs in a more efficient and effective way. We believe this study can be extended to other MASs, especially MASs whose neighborhood relationships are also defined by interaction radius.

## Supporting information

S1 Fig Three-dimensional plot for soft control performance with respect to *r* for different *θ*_*s*_ based on the Vicsek model with *ρ*_*n*_ = 1, *M* = 15, *v* = 0.03 and *η* = 0.1. The simulation terminates when 1 − *ϕ*(*t*) ≤ 10^−3^.
(EPS)

S2 Fig The soft control performance for different *θ*_*s*_ (*θ*_*s*_ = *π*/4, *π*/2 and 3*π*/4) with *ρ*_*n*_ = 1 and *M* = 15. (A) is for the *fixed-heading-shill* scenario; (B) is for the *evolvable-heading-shill* scenario.
(EPS)

S3 Fig The critical point *r*_*c*_0__ varies with different *θ*_*s*_ in the *evolvable-heading-shill* scenario. (A) is the pattern for the comparison of the soft control performance between 25 *centered* shills and 25 times of one single shill in the *evolvable-heading-shill* scenario. (B) is the *r* − *θ*_*s*_ projection of (A). Soft control performance is measured as the average of 500 runs on random position distributions of normal agents.
(EPS)

S1 Appendix Mean-field analysis for the *distributed* strategy soft control performance in the Vicsek model. (PDF)

S2 Appendix Simulations and analysis for the soft control performance based on the linearized Vicsek model. (PDF)

S1 File This file contains the data used in figures. (7Z)
