# Supplementary material for: How does the interaction radius affect the performance of intervention on collective behavior?
Source: PLoS One. 2018 Feb 15;13(2):e0192738. doi: 10.1371/journal.pone.0192738 (PMC5813976; doi:10.1371/journal.pone.0192738)
Supplement: S1 Appendix — (PDF) [file pone.0192738.s004.pdf]

S1 Appendix: Mean-field analysis for the *distributed* strategy soft control performance in the Vicsek model

Caiyun Wang[1,2], Jing Han[1,2*]

1 LSC, Academy of Mathematics and Systems Science, Chinese Academy of Sciences, Beijing 100190, China
2 School of Mathematical Sciences, University of Chinese Academy of Sciences, Beijing 100049, China

*hanjing@amss.ac.cn

Fixed-heading-shill scenario

The updating rule (without the noise) of the heading of normal agents, i.e.,

$$\theta_k(t+1) = \arctan\left(\frac{\sum\limits_{j\in N_k(t)} \sin(\theta_j(t))}{\sum\limits_{j\in N_k(t)} \cos(\theta_j(t))}\right), \quad \text{for} \;\; 1 \leq k \leq n \tag{1}$$

can be written as the following form:

$$e^{i\theta_k(t+1)} = \frac{\sum\limits_{j\in N_k(t)} e^{i\theta_j(t)}}{\|\sum\limits_{j\in N_k(t)} e^{i\theta_j(t)}\|} \text{ for } k = 1, \cdots, n, \tag{2}$$

where i is the imaginary unit. Suppose each normal agent has $\pi r^2 \rho_n$ normal agent neighbors and $\pi r^2 \rho_s$ shill neighbors, where ρ_n is the density of normal agents and ρ_s is the density of shills. Therefore, for normal agent k, we have:

$$e^{i\theta_k(t+1)} \approx \frac{\pi r^2 \rho_n e^{i\theta_k(t)} + \pi r^2 \rho_s e^{i\theta_s}}{\|\pi r^2 \rho_n e^{i\theta_k(t)} + \pi r^2 \rho_s e^{i\theta_s}\|}. \tag{3}$$

Furthermore,

$$e^{i(\theta_k(t+1)-\theta_s)} \approx \frac{\pi r^2 \rho_n e^{i(\theta_k(t)-\theta_s)} + \pi r^2 \rho_s}{\|\pi r^2 \rho_n e^{i\theta_k(t)} + \pi r^2 \rho_s e^{i\theta_s}\|}. \tag{4}$$

It follows from Eq (4) and the Euler's formula that

$$\begin{aligned}\cos\left(\theta_k(t+1) - \theta_s\right) &+ i\sin\left(\theta_k(t+1) - \theta_s\right) \\ &\approx \frac{\pi r^2 \rho_n \cos\left(\theta_k(t) - \theta_s\right) + \pi r^2 \rho_s + i\sin\left(\theta_k(t) - \theta_s\right)}{\sqrt{\pi^2 r^4 {\rho_n}^2 + \pi^2 r^4 {\rho_s}^2 + 2\pi^2 r^4 \rho_n \rho_s \cos\left(\theta_k(t) - \theta_s\right)}}.\end{aligned} \tag{5}$$

According to the nature of the imaginary, we have:

$$\cos\left(\theta_k(t+1) - \theta_s\right) \approx \frac{\pi r^2 \rho_n \cos\left(\theta_k(t) - \theta_s\right) + \pi r^2 \rho_s}{\sqrt{\pi^2 r^4 {\rho_n}^2 + \pi^2 r^4 {\rho_s}^2 + 2\pi^2 r^4 \rho_n \rho_s \cos\left(\theta_k(t) - \theta_s\right)}}. \tag{6}$$

Furthermore,

$$\begin{aligned}1-&\cos^2(\theta_k(t+1)-\theta_s)\\&\approx \frac{\pi r^2{\rho_n}^2}{\pi r^2{\rho_n}^2+\pi r^2{\rho_s}^2+2\pi r^2\rho_n\pi r^2\rho_s\cos(\theta_k(t)-\theta_s)}[1-\cos^2(\theta_k(t)-\theta_s)]\\&=\frac{\rho_n^2}{\rho_n^2+\rho_s^2+2\rho_n\rho_s\cos(\theta_k(t)-\theta_s)}[1-\cos^2(\theta_k(t)-\theta_s)]\end{aligned} \quad (7)$$

For convenience, let $\zeta(t)=1-\cos^2(\theta_k(t)-\theta_s)$. Then, when $|\theta_0-\theta_s|<\pi/2$, direct calculations show

$$\frac{\rho_n^2}{\rho_n^2+\rho_s^2+2\rho_n\rho_s}\zeta(t)<\zeta(t+1)<\frac{\rho_n^2}{\rho_n^2+\rho_s^2+2\rho_n\rho_s\cos(\theta_0-\theta_s)}\zeta(t). \quad (8)$$

Furthermore,

$$\left(\frac{\rho_n^2}{\rho_n^2+\rho_s^2+2\rho_n\rho_s}\right)^{t+1}\zeta(0)<\zeta(t+1)<\left(\frac{\rho_n^2}{\rho_n^2+\rho_s^2+2\rho_n\rho_s\cos(\theta_0-\theta_s)}\right)^{t+1}\zeta(0). \quad (9)$$

Let $A=\frac{\rho_n^2}{\rho_n^2+\rho_s^2+2\rho_n\rho_s\cos(\theta_0-\theta_s)}$ and $B=\frac{\rho_n^2}{\rho_n^2+\rho_s^2+2\rho_n\rho_s}$, through equation (9), the convergent time step T satisfies [1]:

$$\log_A\frac{1-\cos^2(10^{-4})}{1-\cos^2(\theta_0-\theta_s)}<T<\log_B\frac{1-\cos^2(10^{-4})}{1-\cos^2(\theta_0-\theta_s)} \quad (10)$$

Therefore,

$$\max T-\min T=\left(\frac{1}{\ln B}-\frac{1}{\ln A}\right)\ln\frac{1-\cos^2(10^{-4})}{1-\cos^2(\theta_0-\theta_s)} \quad (11)$$

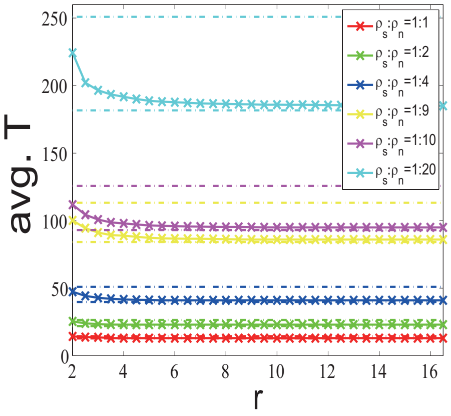

Fig 1. Simulation results *v.s.* mean-field results for different density ratios between shills and normal agents in the *fixed-heading* scenario. $\rho_n=1$, $l=25$, $v=0.03$, $\eta=0$, $\theta_0=0$ and $\theta_s=\pi/4$. Dotted lines represent corresponding upper and lower bounds calculated by mean-field analysis. The simulation results are measured as the average of 100 runs on random position distributions of normal agents[1].

Figure 1 shows the comparison between simulation results and mean-field results for different density ratios between shills and normal agents in the *fixed-heading* scenario. The

[1] The system without noise is regarded as reaching consensus when $\max_{1\le k\le\tilde{n}}\theta_k(t)-\min_{1\le k\le\tilde{n}}\theta_k(t)\le 10^{-4}$.

corresponding numerical values are given in Table 1. In general, the mean-field results conform to the simulation results especial for the lower bounds. And $\max T - \min T$ is relatively small. It proves that r does not have significant influence on the soft control performance in the *fixed-heading* scenario.

Table 1. Simulation values v.s. mean-field value

	simulation results			mean-field results			simulation results : mean-field results		
$\rho_s : \rho_n$	upper bound	lower bound	range	upper bound	lower bound	range	upper bound	lower bound	range
1:1	13	14.27	1.27	12.78	14.44	1.65	0.99	1.02	0.77
1:2	22.96	25.39	2.43	21.86	26.40	4.54	0.96	1.05	0.53
1:4	41	47.29	6.29	39.72	50.96	11.23	0.92	1.03	0.56
1:9	86	100.12	14.12	84.13	113.23	29.10	0.88	1.02	0.49
1:10	95	111.8	16.8	93.00	125.73	32.73	0.89	1.02	0.51
1:20	185	224.3	39.3	181.67	250.90	69.23	0.89	1.02	0.57

Evolvable-heading-shill scenario

Similarly, for each normal agent, suppose it has $\pi r^2 \rho_n$ normal agent neighbors and $\pi r^2 \rho_s$ shill neighbors. Through the updating rule (without noise) of the heading of normal agents, i.e., Eq 1, we have

$$\begin{aligned} \theta_k(1) &\approx \arctan \frac{\pi r^2 \rho_n \sin\theta_0 + \pi r^2 \rho_s \sin\theta_s}{\pi r^2 \rho_n \cos\theta_0 + \pi r^2 \rho_s \cos\theta_s} \\ &= \arctan \frac{\rho_n \sin\theta_0 + \rho_s \sin\theta_s}{\rho_n \cos\theta_0 + \rho_s \cos\theta_s} \end{aligned} \quad (12)$$

Therefore,

$$\Delta\theta \approx \arctan \frac{\rho_n \sin\theta_0 + \rho_s \sin\theta_s}{\rho_n \cos\theta_0 + \rho_s \cos\theta_s}. \quad (13)$$

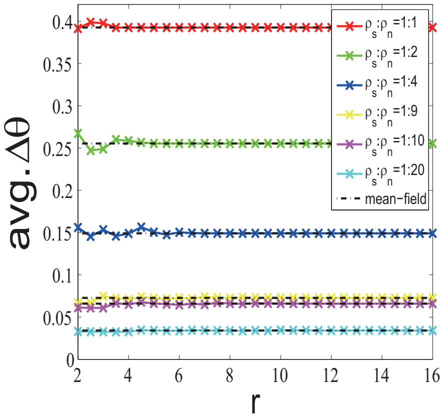

Fig 2. Simulation results *v.s.* mean-field results for different density ratios between shills and normal agents in the *evolvable-heading* scenario. $\rho_n = 1$, $l = 25$, $v = 0.03$, $\eta = 0$, $\theta_0 = 0$ and $\theta_s = \pi/4$. The simulation results are measured as the average of 100 runs on random position distributions of normal agents.

Figure 2 shows the result of comparisons between simulation results and corresponding mean-field results for different density ratios between shills and normal agents in the *evolvable-heading* scenario. Mean-field results are roughly in accordance with simulation

results especially. So it proves that r does not have significant influence on the soft control performance in the *evolvable-heading* scenario.

We can see from Fig. 5D of the main text that: for $\theta_s > \pi/2$, increasing θ_s decreases $\Delta\theta$. This means a larger perturbation perturbs less the system. We will give mean-field proof for this counter-intuitive phenomena in the following.

By Eq (13), regard $\Delta\theta$ as a function of θ_s, then we have:

$$\frac{\mathrm{d}\Delta\theta}{\mathrm{d}\theta_s} = \frac{{\rho_s}^2 + \rho_s\rho_n\cos(\theta_s - \theta_0)}{(\rho_n\sin\theta_0 + \rho_s\sin\theta_s)^2} \tag{14}$$

Therefore, when $\theta_s > \theta_0 + \arccos(-\rho_s/\rho_n)$, $\Delta\theta$ decreases with the increase of θ_s. Especially, $\rho_s : \rho_n$ is set to be $1 : 9$ in the Fig. 5D of the main text, when $\theta_s > \theta_0 + \arccos(-\rho_s/\rho_n) \approx 0.5354\pi$, $\Delta\theta$ decreases with the increase of θ_s. So our mean-field proof for this counter-intuitive critical point is consistent with the simulation result.
