# Supplementary material for: How does the interaction radius affect the performance of intervention on collective behavior?
Source: PLoS One. 2018 Feb 15;13(2):e0192738. doi: 10.1371/journal.pone.0192738 (PMC5813976; doi:10.1371/journal.pone.0192738)
Supplement: S2 Appendix — (PDF) [file pone.0192738.s005.pdf]

S2 Appendix: Simulations and analysis for the soft control performance based on teh linearized Vicsek model

Caiyun Wang[1,2], Jing Han[1,2*]

1 LSC, Academy of Mathematics and Systems Science, Chinese Academy of Sciences, Beijing 100190, China
2 School of Mathematical Sciences, University of Chinese Academy of Sciences, Beijing 100049, China

* hanjing@amss.ac.cn

For the linearized Vicsek model, we first consider the case of the absolute value of the velocities and the noise are all set to be zero. We find in simulations[1] of this simplified linearized Vicsek model (Fig. 1) are similar to patterns that we have found in the Vicsek model.

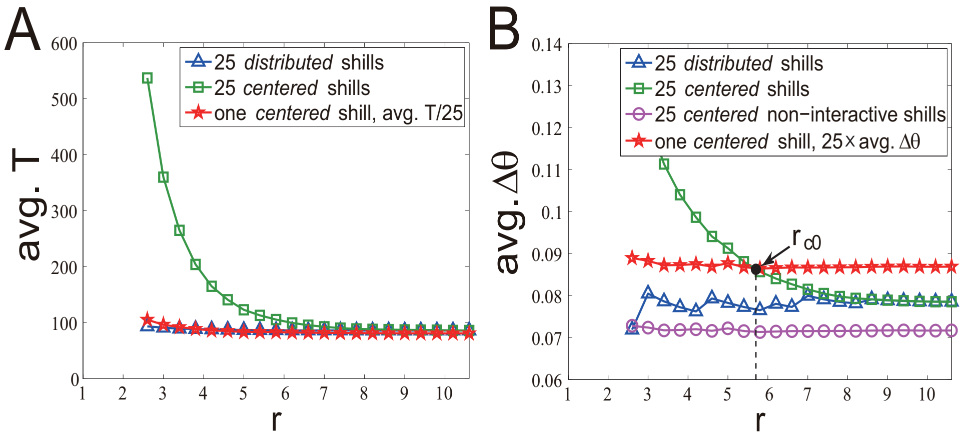

Fig 1. Soft control performance of different strategies in different scenarios based on the linearized Vicsek model. $\rho_n = 1$, $M = 15$, $\theta_s = \pi/4$, $v = 0$ and $\eta = 0$ (A) is the pattern for the *fixed-heading-shill* scenario. (B) is the pattern for the *evolvable-heading-shill* scenario.

We will give theoretical analysis for these patterns in the *evolvable-heading-shill* scenario in the following. In the *evolvable-heading-shill* scenario, the heading of all agents (including normal agents and shills) are updated as follows:

$$\mathbf{\Theta}(t) = A\mathbf{\Theta}(t-1),$$

[1]In simulations, the system without noise is regarded as reaching consensus when $\max_{1\le k\le \tilde{n}} \theta_k(t) - \min_{1\le k\le \tilde{n}} \theta_k(t) \le 10^{-4}$.

where $\mathbf{\Theta}(t) = (\theta_1(t), \theta_2(t), \ \cdots, \ \theta_{n+l}(t))^T$. And

$$A = \begin{bmatrix} \frac{b_{11}}{\sum_{j=1}^{n+l} b_{1j}} \cdots \frac{b_{1n}}{\sum_{j=1}^{n+l} b_{1j}} \cdots \frac{b_{1,n+l}}{\sum_{j=1}^{n+l} b_{1j}} \\ \vdots \\ \frac{b_{n1}}{\sum_{j=1}^{n+l} b_{nj}} \cdots \frac{b_{nn}}{\sum_{j=1}^{n+l} b_{nj}} \cdots \frac{b_{n,n+l}}{\sum_{j=1}^{n+l} b_{nj}} \\ \vdots \\ \frac{b_{n+l,1}}{\sum_{j=1}^{n+l} b_{n+l,j}} \cdots \frac{b_{n+l,n}}{\sum_{j=1}^{n+l} b_{n+l,j}} \cdots \frac{b_{n+l,n+l}}{\sum_{j=1}^{n+l} b_{n+l,j}} \end{bmatrix},$$

where $b_{ij} = 1$ if agent i and agent j are neighbors; $b_{ij} = 0$, otherwise.

Agents will reach consensus, i.e., $\lim\limits_{t\to\infty} A^t\mathbf{\Theta}(0) = (\theta', \theta', \cdots, \theta')^T$, as long as matrix A is a nonnegative primitive statistic matrix [1]. θ' can be calculated as: $\theta' = Y\mathbf{\Theta}(0)$, where Y is the left eigenvector of A with eigenvalue 1 and $Y(1, 1, \cdots, 1)^T = 1$, Y has the form as follows:

$$Y = (\frac{\sum_{j=1}^{n+l} b_{1j}}{\sum_{i=1}^{n+l}\sum_{j=1}^{n+l} b_{ij}}, \frac{\sum_{j=1}^{n+l} b_{2j}}{\sum_{i=1}^{n+l}\sum_{j=1}^{n+l} b_{ij}}, \cdots, \frac{\sum_{j=1}^{n+l} b_{n+l,j}}{\sum_{i=1}^{n+l}\sum_{j=1}^{n+l} b_{ij}}).$$

We can get:

$$\begin{aligned} \Delta\theta &= \theta' - \theta_0 \\ &= \frac{\sum_{i=1}^{n}\sum_{j=1}^{n+l} b_{ij}\left(\theta_i(0) + \theta_j(0) - 2\theta_0\right) + \sum_{i=n+1}^{n+l}\sum_{j=n+1}^{n+l} b_{ij}\left(\theta_i(0) - \theta_0\right)}{\sum_{i=1}^{n+l}\sum_{j=1}^{n+l} b_{ij}}. \end{aligned}$$

For simplified linearized Vicsek model $\theta_i(0) = \theta_0$ for $i = 1, 2, ..., n$ and $\theta_i(0) = \theta_s$ for $i = n+1, ..., n+l$, so

$$\begin{aligned} \Delta\theta &= \frac{(\sum_{i=1}^{n}\sum_{j=n+1}^{n+l} b_{ij} + \sum_{i=n+1}^{n+l}\sum_{j=n+1}^{n+l} b_{ij})(\theta_s - \theta_0)}{\sum_{i=1}^{n}\sum_{j=1}^{n} b_{ij} + \sum_{i=1}^{n}\sum_{j=n+1}^{n+l} b_{ij} + \sum_{i=n+1}^{n+l}\sum_{j=1}^{n} b_{ij} + \sum_{i=n+1}^{n+l}\sum_{j=n+1}^{n+l} b_{ij}} \\ &= \frac{\sum_{i=n+1}^{n+l}(\sum_{j=1}^{n} b_{ij} + \sum_{j=n+1}^{n+l} b_{ij})(\theta_s - \theta_0)}{\sum_{i=1}^{n}\sum_{j=1}^{n} b_{ij} + 2\sum_{i=n+1}^{n+l}\sum_{j=1}^{n} b_{ij} + \sum_{i=n+1}^{n+l}\sum_{j=n+1}^{n+l} b_{ij}} \end{aligned}$$

Because normal agents are randomly placed, all normal agents and shills have the same number of neighboring normal agents, no matter where they are located. Similarly, all shills have similar number of shill neighbors. So let $\hat{n}_n$ represent the number of normal agent neighbors of one agent (including normal agents and shills) and $\hat{n}_s$ represent the number of shill neighbors of one shill. And let ρ_n represent the density of normal agent, i.e., $\rho_n = n/M^2$. We have $\hat{n}_n(r) \approx \pi r^2 \rho_n$. So

$$\Delta\theta = \frac{l(\hat{n}_n + \hat{n}_s)(\theta_s - \theta_0)}{(n+2l)\hat{n}_n + l\hat{n}_s}. \tag{1}$$

By using equation (1), we can explain results obtained from Fig 1B:

(1) **Adding l *distributed* shills ($1 \le \hat{n}_s \le l$):**

Let $\Delta\theta_d$ represent $\Delta\theta$ of the *distributed* strategy, $\Delta\theta_d$ has the same form of equation (1):

$$\Delta\theta_d = \frac{l(\hat{n}_n + \hat{n}_s)(\theta_s - \theta_0)}{(n+2l)\hat{n}_n + l\hat{n}_s}. \tag{2}$$

$\Delta\theta_d$ decreases with the increase of $\hat{n}_n$ and it increases with the increase of $\hat{n}_s$. At the same time, $\hat{n}_n$ increases with the increase of r (black line in Fig. 2); $\hat{n}_s$ increases with the increase of r and it remains constant locally (purple line in Fig. 2). So $\Delta\theta_d$ varies little with the increase of r.

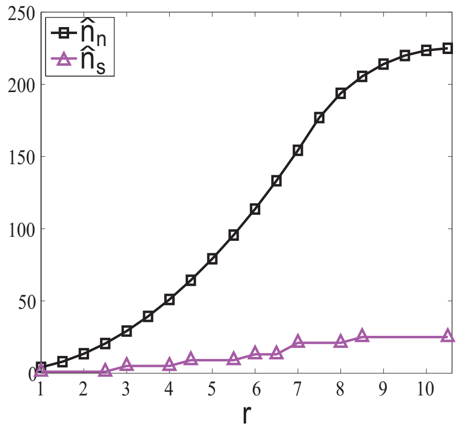

Fig 2. Patterns for $\hat{\mathbf{n}}_\mathbf{n}$ and $\hat{\mathbf{n}}_\mathbf{s}$ with the change of r as the average of 100 runs on random position distributions of 225 normal agents and 25 *distributed* shills with $\mathrm{M} = \mathbf{15}$.

(2) **Adding l *centered* shills ($\hat{n}_s = l$):**

Let $\Delta\theta_c$ represent $\Delta\theta$ of the *centered* strategy. Arise from equation (1), $\Delta\theta_c$ has the following form:

$$\Delta\theta_c = \frac{l(\hat{n}_n + l)(\theta_s - \theta_0)}{(n+2l)\hat{n}_n + l^2}. \tag{3}$$

$\Delta\theta_c$ decreases with the increase of $\hat{n}_n$ and $\hat{n}_n$ increases with the increase of r. So $\Delta\theta_c$ decreases with the increase of r.

(3) **Comparing the *distributed* strategy and the *centered* strategy**:

By comparing equation (2) and equation (3), we have:

$$\Delta\theta_c - \Delta\theta_d = \frac{l\hat{n}_n(n+l)(l-\hat{n}_s)(\theta_s - \theta_0)}{[(n+2l)\hat{n}_n + l^2][(n+2l)\hat{n}_n + l\hat{n}_s]} \geq 0, \tag{4}$$
and $\Delta\theta_c - \Delta\theta_d = 0$ if and only if $\hat{n}_s = l$.

So the *centered* strategy outperforms the *distributed* strategy.

(4) **Comparing the *centered* (interactive) strategy and the *centered* non-interactive case ($\hat{n}_s = 1$)**:

Let $\Delta\theta_n$ represent $\Delta\theta$ of the *centered* non-interactive case, arise from equation (1), $\Delta\theta_n$ has following form:

$$\Delta\theta_n = \frac{l(\hat{n}_n + 1)(\theta_s - \theta_0)}{(n+2l)\hat{n}_n + l}. \tag{5}$$

By comparing equation (3) and equation (5), we have:

$$\Delta\theta_c - \Delta\theta_n = \frac{l\hat{n}_n(n+l)(l-1)(\theta_s - \theta_0)}{[(n+2l)\hat{n}_n + l^2][(n+2l)\hat{n}_n + l]} > 0. \tag{6}$$

So the *centered* interactive strategy outperforms *centered* non-interactive case.

(5) **Comparing the case of adding l *centered* shills and the case of adding one *centered* shill**:

Let $\Delta\theta_o$ represent $\Delta\theta$ of the adding one *centered* shill case, arise from equation (1), $\Delta\theta_o$, has the following form:

$$\Delta\theta_o = \frac{(\hat{n}_n + 1)(\theta_s - \theta_0)}{(n+2)\hat{n}_n + 1}. \tag{7}$$

By comparing equation (3) and l times of equation (7), we have:

$$\Delta\theta_c - l\Delta\theta_o = \frac{[2l\hat{n}_n^2 + (l^2 + l - nl)\hat{n}_n + l^2](1-l)(\theta_s - \theta_0)}{[(n+2)\hat{n}_n + 1][(n+2l)\hat{n}_n + l^2]}. \tag{8}$$

Inferred by equation (8) and $l \ll n$, the number of neighboring normal agents when $r = r_{c0}$ (i.e., $\hat{n}_n(r_{c0})$) can be calculated by:

$$\hat{n}_n(r_{c0}) = \frac{(nl - l - l^2) + \sqrt{l^4 - (6+2n)l^3 + (n^2 - 2n + 1)l^2}}{4l}. \tag{9}$$

The value of r_{c0} can be inferred by the value of $\hat{n}_n(r_{c0})$. At the same time, we have r_{c0} is constant of different θ_s for the simplified linearized Vicsek model case.

What if the absolute value of the velocities is not *zero*? We can prove that in the *evolvable-heading-shill* scenario if the absolute value of the velocities is small enough, the neighborhood graph $G = (V, E)$ will be static during evolution. We first give the following theorem based on the theorem of Liu et al. [2]:

Theorem 1. *For the linearized Vicsek model without noise, let $G_0 = (V, E_0)$ be the initial neighborhood graph. Then, the neighborhood graph is static during evolution, i.e., $G_t = G_0, \forall t \geq 0$, if the absolute value of the velocities v satisfies the following inequality:*

$$v \leq \min\left(\frac{d_1}{\Delta_0}\left(\frac{1}{\tilde{n}}\right)^{\tilde{n}}, \frac{d_2}{\Delta_0}\left(\frac{1}{\tilde{n}}\right)^{\tilde{n}}\right), \tag{10}$$

where $d_1 = r - \max\limits_{i,j \in E_0} d_{ij}(0)$, $d_2 = \min\limits_{i,j \notin E_0} d_{ij}(0) - r$, $\Delta_0 = \max\limits_{i,j}(\theta_i(0) - \theta_j(0))$ and $d_{ij}(t)$ is the distance of agent i and j at time t.

Proof. For any two agents i and j, we have

$$(\cos\theta_i(t) - \cos\theta_j(t))^2 + (\sin\theta_i(t) - \sin\theta_j(t))^2 = 4\left|\sin\frac{\theta_i(t) - \theta_j(t)}{2}\right|^2.$$

Thus, by the properties of Euclidean norm, we have

$$d_{ij}(t) - v\left|\theta_i(t) - \theta_j(t)\right| \leq d_{ij}(t+1) \leq d_{ij}(t) + v\left|\theta_i(t) - \theta_j(t)\right|.$$

That means

$$d_{ij}(t) - v\Delta_t \leq d_{ij}(t+1) \leq d_{ij}(t) + v\Delta_t, \quad t \geq 0 \tag{11}$$

where $\Delta_t = \max\limits_{i,j}(\theta_i(t) - \theta_j(t))$. By the update rule of the heading of normal agent (the equation (3) of the manuscript), we have Δ_t is a non-increasing sequence of time t.

1. **We prove that neighboring agents at $t = 0$ are still neighbors at $t \in [k\tilde{n}, (k+1)\tilde{n})$.**

   In other words, we want to prove:

$$d_{ij}(t) \leq r, \quad \forall i, j \in E_0, \forall t \in [k\tilde{n}, (k+1)\tilde{n}). \tag{12}$$

   We use mathematical induction to prove it.

   First, for $k = 0$.

   For arbitrary $i, j \in E_0$, using (11) and the monotonicity of Δ_t, we have

$$d_{ij}(t+1) \leq d_{ij}(0) + v\sum_{m=0}^{t}\Delta_m \leq d_{ij}(0) + (t+1)v\Delta_0, \quad 0 \leq t \leq \tilde{n} - 1.$$

Moreover, by the condition of v:

$$v \leq \min\left(\frac{d_1}{\Delta_0}\left(\frac{1}{\tilde{n}}\right)^{\tilde{n}}, \frac{d_2}{\Delta_0}\left(\frac{1}{\tilde{n}}\right)^{\tilde{n}}\right), \tag{13}$$

we can get

$$d_{ij}(t+1) \leq d_{ij}(0) + (t+1)\frac{d_1}{\Delta_0}\left(\frac{1}{\tilde{n}}\right)^{\tilde{n}}\Delta_0 < r, \quad 0 \leq t \leq \tilde{n}-1.$$

This means that those agents that are neighbors at $t=0$ that are still neighbors at $t \in [0, \tilde{n})$.

Second, we assume (12) hold for all $k \leq K$. By (11) and the result of Liu et al. [2]: $\Delta_t \leq L^h \Delta_0,$ where $\forall t \in [h\tilde{n}, (h+1)\tilde{n})$ with $h \leq K+1$ and $L = 1 - \tilde{n}(\frac{1}{\tilde{n}})^{\tilde{n}}$, for any $i, j \in E_0$ and $t \in [(K+1)\tilde{n}, (K+2)\tilde{n})$ we have

$$\begin{aligned}
d_{ij}(t) &\leq d_{ij}(t-1) + v\Delta_t \leq d_{ij}(0) + v\sum_{m=0}^{t-1}\Delta_l \\
&\leq d_{ij}(0) + \tilde{n}v(1 + L + L^2 + \cdots + L^{K+1})\Delta_0 \\
&< d_{ij}(0) + \tilde{n}v\frac{1}{1-L}\Delta_0 \\
&\leq d_{ij}(0) + \tilde{n}\frac{d_1}{\Delta_0}\left(\frac{1}{\tilde{n}}\right)^{\tilde{n}}\frac{1}{1-L}\Delta_0 \leq r.
\end{aligned}$$

Hence, those agents that are neighbors at $t=0$ will be neighbors for $t \in [(K+1)\tilde{n}, (K+2)\tilde{n})$. Therefore, (12) is true when $k = K+1$.

2. **We prove that those agents which are not neighbors at $t=0$ that will never be neighbors at $t \in [k\tilde{n}, (k+1)\tilde{n})$.**

In other words, we want to prove:

$$d_{ij}(t) > r, \quad \forall i, j \notin E_0, \forall t \in [k\tilde{n}, (k+1)\tilde{n}) \tag{14}$$

We use mathematical induction to prove it.

First, for $k=0$.

For arbitrary $i, j \in E_0$, using (11) and the monotonicity of Δ_t, we have

$$d_{ij}(t+1) \geq d_{ij}(0) - v\sum_{m=0}^{t}\Delta_m \geq d_{ij}(0) - (t+1)v\Delta_0, \quad 0 \leq t \leq \tilde{n}-1.$$

Moveover, by (13), we can get

$$d_{ij}(t+1) \geq d_{ij}(0) - (t+1)\frac{d_2}{\Delta_0}\left(\frac{1}{\tilde{n}}\right)^{\tilde{n}}\Delta_0 > r, \quad 0 \leq t \leq \tilde{n}-1.$$

This means that those agents that are not neighbors at $t=0$ that are not neighbors at $t \in [0, \tilde{n})$.

Second, we assume (14) hold for all $k \leq K$. Similarly, by (11) and the result of Liu et al. [2]: $\Delta_t \leq L^h \Delta_0,$ where $\forall t \in [h\tilde{n}, (h+1)\tilde{n})$ with $h \leq K+1$ and $L = 1 - \tilde{n}(\frac{1}{\tilde{n}})^{\tilde{n}}$,

for any $i, j \in E_0$ and $t \in [(K+1)\tilde{n}, (K+2)\tilde{n})$ we have

$$
\begin{aligned}
d_{ij}(t) &\geq d_{ij}(t-1) - v\Delta_t \geq d_{ij}(0) - v\sum_{m=0}^{t-1}\Delta_l \\
&\geq d_{ij}(0) - \tilde{n}v(1 + L + L^2 + \cdots + L^K)\Delta_0 \\
&> d_{ij}(0) - \tilde{n}v\frac{1}{1-L}\Delta_0 \\
&\geq d_{ij}(0) - \tilde{n}\frac{d_2}{\Delta_0}\left(\frac{1}{\tilde{n}}\right)^{\tilde{n}}\frac{1}{1-L}\Delta_0 \geq r.
\end{aligned}
$$

Hence, those agents that are not neighbors at $t = 0$ will not be neighbors for $t \in [K\tilde{n}, (K+1)\tilde{n})$. Therefore, (14) is true when $k = K + 1$.

□

From Theorem 1, we can see that if v satisfies Eq (13), these results what we have gotten in the case of $v = 0$ are also hold based on the linearized Vicsek model without noise.

References

1. Seneta E. Non-negative matrices and Markov chains. Springer Science & Business Media; 2006 Jul 2.

2. Liu Z, Guo L. Connectivity and synchronization of Vicsek model. Science in China Series F: Information Sciences. 2008 Jul 1;51(7):848–858.
